# Language control in bilingual language comprehension: evidence from the maze task

**DOI:** 10.3389/fpsyg.2015.01179

**Published:** 2015-08-21

**Authors:** Xin Wang

**Affiliations:** Department of Education, University of OxfordOxford, UK

**Keywords:** code-switching, language switching, bilingual language comprehension, switch cost, inhibitory control, language dominance, bilingualism

## Abstract

Most empirical evidence on switch costs is based on bilingual production and interpreted as a result of inhibitory control. It is unclear whether such a top–down control process exists in language switching during comprehension. This study investigates whether a non-lexical switch cost is involved in reading code-switched sentences and its relation to language dominance with cross-script bilingual readers. A maze task is adopted in order to separate top–down inhibitory effects, from lexical effects driven by input. The key findings are: (1) switch costs were observed in both L1–L2 and L2–L1 directions; (2) these effects were driven by two mechanisms: lexical activation and inhibitory control; (3) language dominance modulated the lexical effects, but did not affect the inhibitory effects. These results suggest that a language control mechanism is involved in bilingual reading, even though the control process is not driven by selection as in production. At the theoretical level, these results lend support for the Inhibitory Control model during language switching in comprehension; while the BIA/BIA+ model needs to incorporate a top–down control mechanism to be able to explain the current findings.

## Introduction

Code-switching (CS) is a common and natural occurrence in multilingual societies which, due to language contact, has existed for centuries. Like other linguistic behavior, CS is not a haphazard occurrence but is in various ways governed by rules and linguistic constraints (Muysken, 2004). Because CS involves a switch from one language to another, many psycholinguistics researchers have investigated whether this process of switching incurs processing costs (e.g., Heredia and Altarriba, 2001). Most of this research has focused on bilingual production. Studies have demonstrated significantly slower reaction times (RTs) when bilinguals switched languages between items in a picture- or number-naming task, compared to when they named non-switched trials (e.g., Meuter and Allport, 1999; Costa and Santesteban, 2004; Costa et al., 2006). That is, when bilinguals switched from one language to the other, a cognitive cost incurred. For instance, Meuter and Allport (1999) presented Spanish–English bilinguals with Arabic numerals and instructed them to name them in either English or Spanish. In a non-switch trial, subjects named the numerals in the same language twice in a row, while a switch trial required them to switch from one language to the other. Their RTs for switch trials were significantly slower than for non-switch trials, suggesting the presence of a switch cost. Even though this type of robust cost was often observed in controlled, cued tasks that involved involuntary language switching, recent evidence from tasks that elicited voluntary language switching in a more natural scenario demonstrates that bilinguals might also need time to switch from one language to the other even when given freedom to name trials without cues (Gollan and Ferreira, 2009; Gollan et al., 2014). These results suggest that such switch costs could also occur during CS in natural conversations.

Another key finding associated with language switching is the role that language dominance plays: switching into the dominant language incurs a greater switch cost than switching into the non-dominant language, i.e., there is a switch cost asymmetry (e.g., Hernandez and Kohnert, 1999; Jackson et al., 2001; Philipp et al., 2007; Verhoef et al., 2009). This asymmetry can be explained by Green’s (1998) Inhibitory Control (IC) model, which specifies a resolution of cross-language competition through an inhibitory mechanism that regulates the bilingual lexico-semantic system for language processes. This is based on the question of how a bilingual can accurately bind external cues with one of their two possible linguistic representations in a given language task. According to the IC model, this is achieved by suppressing lemmas with language tags irrelevant to the task, while activating/selecting lemmas in the target language to communicate. For instance, if a Chinese–English bilingual is asked to complete a picture naming task in English, they will suppress lemmas tagged as Chinese and activate lemmas tagged as English so that 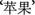 (*mean ‘apple’*) will not compete with ‘APPLE’ for selection. Therefore, if bilinguals intend to switch from one language to the other, they operate control processes to suppress the active language and activate the other language for output. The IC model also predicts that the control process is not effortless and might induce a time lag for language switching. Switching back to the dominant language may incur larger costs, as inhibition of the dominant language takes more executive effort and thus takes longer to overcome in reactivation than the mirroring process in non-dominant language trials. Furthermore, recent neural evidence (e.g., PET, fMRI) suggests that L1 and L2 representations share a common neural network and that competition to control output in language selection is mediated by the left dorsolateral prefrontal cortex (Price et al., 1999; Hernandez et al., 2001; Chee et al., 2003; Abutalebi and Green, 2007), as well as by sub-cortical areas like the left caudate (Abutalebi and Green, 2008; Luk et al., 2011; Wang et al., 2013).

On the other hand, a concern over the IC model to account for switch cost asymmetries is that some studies reported symmetrical switch costs in bilingual production (e.g., Costa and Santesteban, 2004; Costa et al., 2006; Christoffels et al., 2007). This type of evidence suggests that equal inhibition could be applied to both languages or the inhibition mechanism itself is not sufficient (or even wrong) to explain switch costs in production (Finkbeiner et al., 2006; Philipp et al., 2007; Gollan and Ferreira, 2009; Verhoef et al., 2009; Runnqvist et al., 2012; Bobb and Wodniecka, 2013; Declerck and Philipp, 2015). The IC model relies on the notion of persisting inhibition; while alternative accounts to explain switch cost asymmetries favor other mechanisms based on empirical evidence from various paradigms with bilinguals of different levels of proficiency. Common to these accounts is that inhibition is not necessarily involved in language switching; rather, persistent activation or fast–speed lexical selection can explain the switch cost asymmetry (Finkbeiner et al., 2006; Philipp et al., 2007; Runnqvist et al., 2012). In particular, Verhoef et al. (2009) demonstrated that unbalanced Dutch–English bilinguals produced asymmetrical switch costs for short cue-to-stimulus intervals (CSI) but symmetrical switch costs for long CSI. These results indicate that bilinguals could bias the response of the target language through endogenous control given long CSI (i.e., long preparation times). But the long CSI did not benefit the L1 non-switch/repeat trials, suggesting interference of the non-target language influences all trial types except for the L1-repeat trials. They term this effect as “L1-repeat-benefit” for unbalanced bilinguals to account for switch cost asymmetries. It is beyond the scope of this paper to discuss all the alternative accounts for the switch cost asymmetry on the production side, yet, the presence of switch costs, even if symmetric effects, indicates some kind of processing costs associated to alternating between two languages, some of which can be explained by an inhibitory mechanism.

In the domain of language comprehension, similar questions have been investigated to determine the presence of a switch cost and its relation to language dominance (e.g., Grainger and Beauvillain, 1987; Von Studnitz and Green, 1997; Thomas and Allport, 2000; Proverbio et al., 2004; Grainger et al., 2010; Ibáñez et al., 2010). The major debates center on the locus of switch costs in comprehension vs. production and the relevant theoretical models accounting for empirical evidence associated with comprehension vs. production tasks. In production, the switch cost is attributed to the inhibitory control mechanism that operates in a top–down fashion; while a similar effect in comprehension is more likely to be driven by a mechanism that operates reactively to input and context. Given the available comprehension studies, the findings are rather mixed and show different switch cost patterns from language production.

First, one line of research in bilingual visual word recognition demonstrate that both languages are active even when the input is exclusively in one language (e.g., Van Hell and De Groot, 1998; Brysbaert et al., 1999). In particular, the cross-language masked priming paradigm has demonstrated the robust influence of one language on the other even when the bilingual participants were only aware of the target language (Grainger, 1993; Gollan et al., 1997; Jiang and Forster, 2001; Davis et al., 2003; Wang, 2007, 2013; Wang and Forster, 2010). All of this evidence suggests that bilinguals do not selectively activate or deactivate their two languages. Rather, both of a bilingual’s language systems are available during lexical retrieval until the best candidate is selected to match an input word. When tasked with comprehending language switches, a bilingual needs to be able to access the mental representation of the target language (i.e., the switched language) immediately after retrieving the lexical information in the non-target language. This raises the question whether a cognitive cost is incurred in the process of comprehending language switches (and/or code-switches). If so, can the inhibitory control mechanism account for the switch cost pattern in comprehension the way it can in production?

Second, behavioral data of switch costs are not always consistent with ERP (i.e., Event-Related Potential) measures in comprehension. For instance, Ibáñez et al. (2010) showed no evidence of switch costs in reading switched sentences, and Bultena et al. (2014) found switch costs from L1 to L2, but not from L2 to L1; however, ERP measures indicate that such costs do occur (e.g., Moreno et al., 2002; Proverbio et al., 2004; Chauncey et al., 2008; Van Der Meij et al., 2011). In a single word reading task, Chauncey et al. (2008) showed language switching effects in both L1–L2 and L2–L1 directions, as reflected in the N250 ERP component, in a priming paradigm regardless of whether the primes were masked or unmasked to the French–English participants. They interpreted the results as a consequence of greater cognitive efforts exerted in processing French-English prime-target pairs (e.g., *cheveu-loan*, ‘*cheveu*’ means ‘hair’ in English), compared to within-English prime-target ones (e.g., *dust-loan*). Two crucial implications, as argued by the authors, can be drawn from their results: first, the language switching effects in reading unrelated cross-language prime-target pairs indicate language membership information is automatically computed in bilingual reading because unrelated masked primes in L1 or L2 were largely invisible to bilinguals; second, it is unlikely for a control mechanism external to the lexicon, as specified in the IC model, to be able to account for the switch effects when the primes were invisible and the similar effects when the primes were visible. Thus, the language switching effects observed in ERPs were interpreted as the result of inhibiting lexical representations in the non-response language through the language node in the lexicon, as specified in the Bilingual Interactive Activation model (Grainger and Dijkstra, 1992; Van Heuven et al., 1998), when reading from the prime to target (i.e., switching between languages).

Third, evidence of asymmetry in switch cost between the dominant and non-dominant language during language switching in comprehension contrasts with the pattern observed in production. Both Proverbio et al. (2004) and Chauncey et al. (2008) demonstrated a larger switch cost from the dominant language to the non-dominant language, while a larger cost was usually observed when switching to the dominant language in production (e.g., Meuter and Allport, 1999; Costa and Santesteban, 2004). In Bultena et al.’s (2014) study, Dutch–English bilinguals completed a self-paced reading task consisting of sentences that alternated between L1 and L2 right after the main verbs. Switch costs were observed from L1 to L2, but not vice versa (i.e., asymmetry). In addition, Bultena et al. (2014) found that language dominance played a role in switch costs. That is, the magnitude of the costs was correlated with relative proficiency in L2: low proficiency readers took more time to switch. As Bultena et al. (2014) argue, these results are best explained by the relative activation strength of the two languages, rather than an inhibitory control mechanism. The inhibitory account assumes a top–down process where lexical competition at the conceptual level needs to be resolved for output to occur in the target language (Levelt, 1992; Costa et al., 1999; De Bot, 2007). It is less likely for such a mechanism to play a role in bilingual reading, as comprehension is driven by input, implying a bottom–up process at the initial stage. A switch cost in comprehension is more likely to be the result of relative resting-level activation in L1 and L2. L1 lexical representations are easier and faster to activate than L2 representations due to their frequency of usage. Hence, a different pattern is observed in comprehension: switching to L2 incurs a cost due to more effort/time required to activate L2, while switching to L1 yields little cost. This activation account is able to explain the language dominance effect: more effort/time is needed to activate low-proficiency L2 in processing, inducing a larger switch cost.

Thus, the debate remains whether inhibitory control processes play any role in bilingual comprehension of language switches, even though the IC model is assumed to broadly explain how bilinguals select between active representations in both languages through control at different levels of processing. Specifically, the question is whether switch costs in comprehension are driven by a general task control mechanism (i.e., the IC model) or a control mechanism of language activation specific to the lexicon (i.e., the BIA/BIA+ model). The main difference between these two accounts is the locus of switch costs: the IC model attributes the effect to the resolution of competition between two language task schemas; while the BIA/BIA+ model attributes the effect to the modulation of lexical activation within the bilingual lexicon. Further evidence from studies that do not involve language switching suggests that inhibitory processes associated with executive control were present in bilingual language comprehension (Macizo et al., 2010; Pivneva et al., 2014). In Macizo et al.’s (2010) study, Spanish–English bilinguals were instructed to judge whether English words presented in pairs (e.g., *pie-toe*) were semantically related or not. One of the English words was an interlingual homograph (e.g., *pie*, which means *foot* in Spanish). Macizo et al. (2010) found that despite the task being run exclusively in English, the Spanish meaning of these homographs influenced participants’ subsequent processing of their English translation equivalents (e.g., *foot-present*). This suggests that the selection of the appropriate meaning in English involves inhibition of the non-target meaning in Spanish. In addition, Pivneva et al. (2014) found that executive control modulates cross-language activation during L2 sentence reading. To be specific, they found greater executive control among bilinguals but not L2 proficiency reduced cross-language activation in terms of interlingual homograph interference, thus suggesting a role of a domain-general control in bilingual comprehension. As these two studies were done exclusively in one language, the findings appear to be more in line with the idea of a language control mechanism external to the lexicon.

Another motivation to investigate the language control effect in reading is to test whether the BIA/BIA+ model of word recognition would be sufficient to explain bilingual reading. The BIA/BIA+ model of word recognition would predict both bottom–up and top–down processes in bilingual reading. Language membership, which the model represents through a language node, is identified at the word level, relatively late in processing; however, the model also incorporates top–down schemas that steer task-specific processing (Dijkstra and Van Heuven, 2002). In the context of sentence processing, Bultena et al. (2014) argue that the activation of the language nodes can influence the processing of subsequent switch trials through this top–down control mechanism. That is, switching to the target language involves inhibiting the language nodes in the non-target language to allow effective comprehension, and this can induce a processing cost. It is unclear to what extent this top–down control process takes place in bilingual reading. Would an inhibitory control mechanism external to the lexicon be necessary to account for language switching in reading?

## The Current Study

One purpose of the current work is to advance the methodological practice in order to tease apart the lexical effect from the language control effect (non-lexical) during language switching in comprehension. In measuring the switch cost in comprehension, previous studies have always compared the switch trials to the non-switch trials in order to demonstrate the behavioral difference. For instance, in the self-paced reading paradigm adopted by Bultena et al. (2014) comparisons were made between reading non-switch sentences in L2 English (e.g., “*The surprised women bake a pie for their aunt”)* and switch sentences from L2 English to L1 Dutch *(e.g., “The surprised women bake een taart voor hun tante”).* The switch point was always located directly after the verb and the reading times were recorded in a word-by-word fashion. Reading time differences, if there were any, could be observed by comparing ‘*a pie for their aunt’* and *‘een taart voor hun tante.’* If we would expect any switch effects driven by mechanisms external to the lexicon, this measurement is confounded by the stimulus itself (e.g., L2 sentences vs. L1 sentences); naturally, one would expect differences between readings in L1 vs. L2. Therefore, it is hard to tease apart this input-driven lexical effect from other non-lexical effects. This might be the reason why mixed findings of switch costs were reported in the comprehension literature. Ideally, to tease apart the lexical effect in reading a sentence, one needs to find a comparable condition where the same lexical items in one language were either preceded by lexical items in the same language or by their counterparts in the other language. Here, the counterparts should be the lexical items on which bilinguals most likely code-switch in communication. Any robust difference observed on the same input preceded by a non-switch lexical item vs. a switch one can be interpreted as switch effects apart from lexical effects.

**FIGURE 1:**
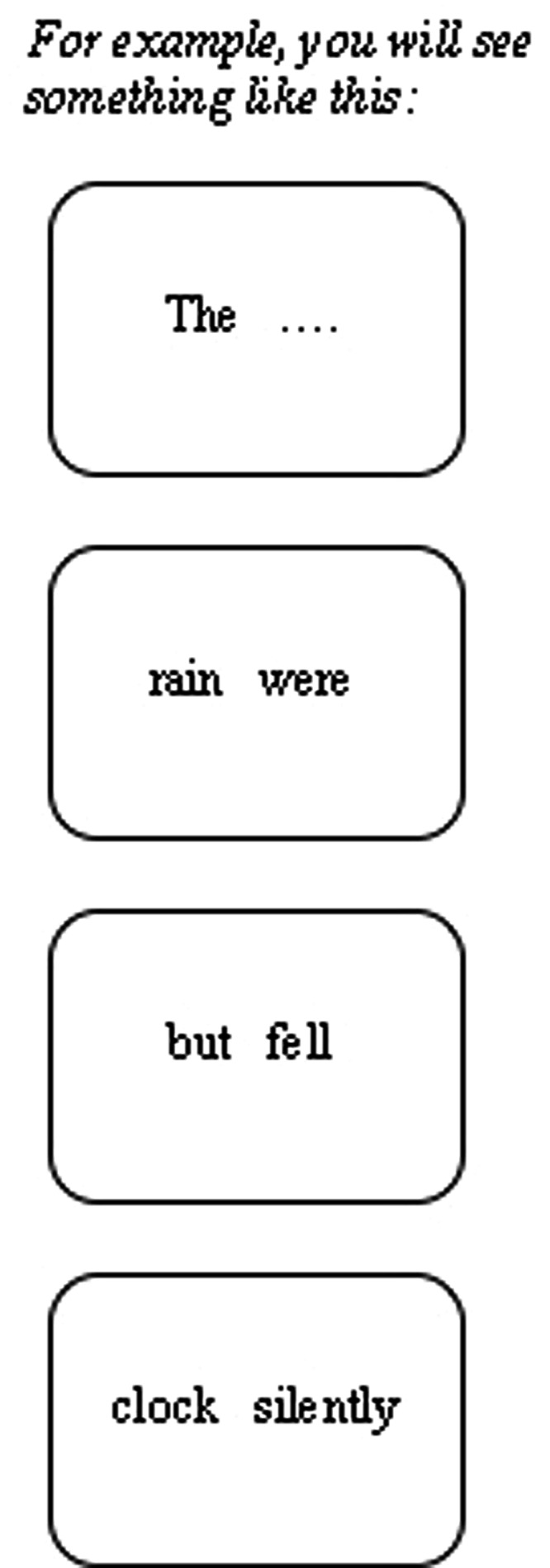
**The rain fell silently**.

This can be achieved by using a different reading paradigm, the maze task (Forster et al., 2009; Forster, 2010). In this task, the objective for the participant is to continue a sentence – from the first word/trial to the last word/trial – by choosing one of two alternatives presented on the computer screen (i.e., a word “maze”). The participant was presented with two words/alternatives at a time, only one of which was grammatically acceptable to continue the sentence. If the participant chose an incorrect word, an error message appeared and a new sentence would begin (see **Figure 1**, more on this in the Materials and Methods section). Empirical evidence suggests that the maze task is sensitive to frequency effects and closely corresponds with data generated from other reading paradigms, such as eye-tracking (Witzel et al., 2012). One advantage of this task, compared to other reading paradigms, is that it forces the reader into a strictly incremental mode of processing with *little spill-over effects* (Forster et al., 2009). Eye-tracking places few restrictions on the way participants approach reading and allows for strategies on any given item in reading a sentence. Therefore, spill-over effects occur if the gaze is shifted to the next word too quickly before it has been completely processed. In a similar way, participants might adopt a strategy whereby they press the button to move on to the next word as soon as they have recognized the word and integrated it into the developing sentence representation in a self-paced reading task. This strategy would buffer each word for reconstruction later in the sentence, leading to spill-over effects. However, the maze task limits these strategies (e.g., ‘wait-and-see’) available to participants by forcing them to process each word carefully enough to continue the sentence. In this way, the maze task has the potential to provide highly localized indications of processing time differences during online sentence comprehension. Thus it should indicate processing time differences at precisely the words predicted to yield such effects. And although processing time differences incurred at a given point in a sentence could influence decisions on subsequent words (i.e., lead to “spillover” effects), empirical evidence shows that these effects were not reliable (Forster et al., 2009; Witzel et al., 2012). The other advantage is that the task cannot be performed unless the sentence is understood. These merits of this paradigm would allow us to measure the same input/word, not being confounded by spill-over effects, by manipulating the preceding words/trials (switch vs. non-switch).

The second purpose is to investigate cross-script switch effects in reading, as distinctive orthographies of a language pair would be more indicative of whether a general task control mechanism is involved in bilingual reading. To identify and process language switches, a within-script reader would need to identify language membership following lexical activation. The BIA/BIA+ model predicts that this process takes place after word identification by activating the language node associated with one of the bilingual’s two languages and inhibiting the other. A bi-script reader (e.g., Chinese–English), however, would be able to identify which language the input belongs to fairly early in processing, because the orthographic features of one language (e.g., Chinese, a logographic language) are quite distinct from the other (e.g., English, an alphabetical language). It is unlikely that the lexical processor would ‘wait’ for the word recognition process to identify language membership; rather, the input would directly cue bilinguals to the language membership. Therefore, the language node does not seem necessary for a bilingual lexicon with two separate orthographies. Rather, the BIA model predicts no non-lexical costs in reading cross-script language switches. Alternatively, the BIA+ model might predict non-lexical switch costs through the task schema; however, it is unlikely these effects are generated from task demands or participants strategies in the maze.

The third goal of this study is to simulate CS in natural communication, by adopting code-switched structures/sentences frequently used among the Chinese–English bilingual community, rather than artificially locating a switch point (more on this in the Materials and Methods section).

Finally, to understand whether/how ‘language dominance’ plays a role in language switching, two groups of Chinese–English bilinguals (Chinese-dominant bilinguals vs. English-dominant bilinguals) will be recruited to test on the same language materials.

## Materials and Methods

### Participants

Participants were English–Chinese bilingual undergraduates or graduates recruited from the National University of Singapore (NUS), upon approval from the NUS-IRB ethics committee. In assessing bilingual dominance, the present study adopted a recent language survey measure, namely the *bilingual dominance scale*, devised by Dunn and Fox Tree (2009) to classify and select bilingual participants (see Appendix A in Supplementary Material). We administered the language dominance scale to 250 students at the NUS, without revealing the purpose of the survey. Eventually, we selected 25 English-dominant bilinguals who scored +15 or above for English after subtracting the scores for Chinese, and 25 Chinese-dominant bilinguals who scored +15 or above for Chinese after subtracting their scores for English. These cut-off values to gage language dominance were suggested in Dunn and Fox Tree (2009). In addition, we only recruited participants who had lived in Singapore for at least 10 years to ensure the consistence of their linguistic environment, as the materials used in the experiment were specifically constructed based on the English–Chinese CS context in Singapore. Further, five more questions (Q12–Q16) were added into the survey for a more accurate assessment of the participants’ linguistic profiles.

### Task

During the maze task, the first word of each sentence was always given, and the participant started choosing the correct word from the second trial onward by pressing the ‘LEFT’ or ‘RIGHT’ key. The ‘LEFT’ key was associated with the word on the left and the ‘RIGHT’ key with the word on the right. The RTs taken to select the correct word at every word/trial of the sentence were recorded by DMDX (Forster and Forster, 2003). **Figure 1** provides an illustration of the task.

### Materials and Design

As this study focuses on the comprehension of CS, it is essential that the CS materials replicate the qualities of natural CS utterances and do not read complex or odd to bilingual participants. A quick review of the CS corpus literature indicates that the predominant English–Chinese CS among the Singaporean community is in functional-lexical phrases, particularly Determiner Phrases (Ong and Zhang, 2010). As such, single-word switches are preferred to phrasal switches among the target bilingual community. This CS pattern is consistent with the Matrix Language Frame (MLF) model proposed by Myers-Scotton (2005). According to the MLF model, the *matrix language* provides the morpho-syntactic frame of a code-switched sentence (e.g., Det + Noun); while the other participating language is known as the *embedded language.* In order to have a systematic measure, we used CS sentences whose matrix language was English, with embedded lexical nouns in Chinese. Instead of choosing the switch point at the phrasal boundary (i.e., the whole DP), we used CS lexical nouns preceded by a determiner in the matrix language. The embedded Chinese nouns were translation equivalents of their counterparts in English.

To ensure that the sentences were natural instances of CS used by the local bilingual community, all of the originally constructed CS sentences were judged by five Singaporean bilinguals, who frequently code-switched between English and Chinese, on a *1–7* Likert scale. They were instructed to read each sentence aloud and then judge the likelihood of producing such utterances in real conversations from 1 to 7, *1* being *extremely rare* and *7* being *extremely frequent*. Only sentences rated above 6 were selected for the study.

A total of 110 sentences were constructed, including 30 filler sentences (15 English sentences and 15 code-switched sentences) and 40 pairs of critical sentences (a switch and a non-switch version of each sentence). Sentences in each pair were identical to each other except that the lexical noun was in Chinese in the switch version. Translation equivalents were used in all the instances of code-switches. All the sentences were constructed so that they were at least five words long, and RTs on three different words/regions of each critical and control sentence were measured. An illustration of this design is presented in **Table 1**.

**Table 1:** Design of code-switching (CS) and non-CS sentences.

Target	Alternative	Region
**Switch mode**		
I	xxx	
polished	thus	
my	drew	1
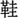	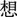	2
yesterday	sad	3
**Non-switch mode**		
I	xxx	
polished	thus	
my	drew	1
shoe	think	2
yesterday	sad	3

The Chinese character 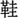 is the translation equivalent of *shoe* and the character 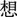 is the translation equivalent of *think*. Subjects would have to select the target word at each step to continue the sentence ‘I polished my shoe/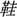 yesterday.’ In order to measure whether processing CS input takes time, the alternative/distracter should be comparable across the switch and non-switch conditions.

In addition, the two alternatives in each trial were unrelated semantically and syntactically and could not be collocated. For instance, if the target word was ‘very,’ then a word like ‘handsome’ would not be a suitable alternative even if the word ‘handsome’ is not a syntactically acceptable option following the preceding word, because the phrase ‘very handsome’ would incur additional processing load/time.

It is important that the point of CS remained unpredictable throughout the experiment. To achieve this, half of the switches occurred at the subject position, while the other half occurred at the object position. Furthermore, a wide range of determiners was selected preceding the CS lexical noun. These included *a, the, this, that, his, her, they, their, my, its, our*, and *some.* A few of the CSs were preceded by the preposition *of* as well.

Two counterbalanced lists were constructed so that the CS sentences appearing on List A would appear as non-CS sentences on List B, and vice versa (see Appendix B). Within each list, there were two conditions (20 switch vs. 20 non-switch sentences), with half of the switches at the subject position and the other half at the object position. Thirty fillers were included in each list so that the participants were not biased toward processing CS noun phrases. The 15 CS fillers switched on lexical adjectives, verbs, or adverbs. In addition, each list included five practice sentences prior to the actual test. An equal number of subjects were randomly assigned to each list for testing.

### Procedure

Each subject was given written instructions that the task was to complete sentences through a maze game, as demonstrated in **Figure 1**, and that the sentences would be completed in a word-by-word procedure. They were aware that each trial would present two alternatives from which they needed to choose the correct one. If they failed to choose the correct one, that sentence would stop and a new sentence would begin. They were asked to respond as accurately and quickly as possible on the maze task, by pressing either the left or right button to continue a sentence.

At the end of the experiment, all subjects were checked on the Chinese characters used in the experiment (see Appendix C), to ensure that they were familiar with the Chinese characters. RTs on three words, or “*Regions*,” were measured for each critical sentence: the code-switched word (the CS, namely, *Region 2*), the word before the CS word (before CS, namely, *Region 1*), and the word after the CS word (after CS, namely, *Region 3*). The same regions were measured in the control sentences.

## Results and Discussion

Among the three measured regions, *Region 3 is critical*, where any behavioral difference between the switch and non-switch conditions should *not* be driven by the input, but due to language switching itself. If there was an inhibitory effect apart from the lexical effect involved in reading CSs, one would expect a significant delay in Region 3 across switch vs. non-switch conditions. The comparison in Region 2 is similar to previous studies (e.g., Bultena et al., 2014), which involves the switched lexical items in the other language. One would expect the effects observed in Region 2 are most likely to be attributed to the lexicon, plus an effect of language switching due to the similar mechanism in Region 3. Therefore, in the current design, one would predict an inhibitory effect observed in Region 3 if the control processes were involved in reading code-switches, as well as lexical effects in Region 2 potentially confounded by inhibitory effects.

### Data Trimming and Statistical Procedure

The experimental design was a factorial 2 × 3 × 2, with Group (2 levels) as a between-subject independent variable and Region (three levels) and Switch Mode (two levels) as within-subject independent variables. In analysing the data, subjects who made more than 10% errors and those who failed to recognize any Chinese character in the post-experiment test were rejected. Six English-dominant bilinguals were rejected because they failed to recognize some of the Chinese characters on the list after the experiment. Another six English-dominant bilinguals were recruited to replace them. They completed a re-run of the experiment, and their data were included in the analysis. Additionally, in trimming the data, RTs lower than 300 ms or higher than 1500 ms were excluded from analysis.

Statistical analyses were performed by fitting a linear mixed effects model to response times (RTs) (Baayen, 2008). Unlike more traditional ANOVAs, mixed-effects models take raw un-averaged data as input and incorporate both random effects of participants and items within a single analysis. The fixed-effect factors were Group (Chinese-dominant and English-dominant), Region (before CS, CS, and after CS), and Switch Mode (switch vs. non-switch). Models were fitted using a restricted maximum likelihood technique. The lmer function from the lme4 package in R was used (version 3.1.0; CRAN project; R Core Team, 2013). *P*-values were derived by Markov Chain Monte Carlo simulation (Baayen et al., 2008); all significant main effects and interactions with *t*-values greater than 2 are reported.

### Data Analysis

As shown in **Table 2**, collapsing all the *Regions*, in error analysis, there was a main effect of *language dominance* (*t* = 4.81, *p* < 0.0001), but neither main effect of *switch mode* (*t* = 0.97, *p* = 0.33), nor interactions between *language dominance* and *switch mode* (*t* = 0.38, *p* = 0.71). This pattern indicates that Chinese-dominant bilinguals made significantly more errors in processing both switch and non-switch English matrix sentences than English-dominant bilinguals. This is expected, as Chinese-dominant bilinguals were less proficient in English.

**Table 2:** Mean reaction times (RTs; in ms) and error rates (in % in parentheses) of English-dominant and Chinese-dominant Bilinguals.

	Non-switch	Switch	Difference
Chinese-Dominant	877 (9.3)	873 (8.5)	4 (0.8)
English-Dominant	731 (3.5)	820 (3.1)	89 (0.4)

In RT mixed-effects analysis, there was a main effect of *language dominance* (*t* = 7.72, *p* < 0.0001), which means that both groups showed different levels of language proficiency in reading sentences, consistent with the error data. In addition, there was a significant interaction between *switch mode* and *language dominance* (*t* = 7.88, *p* < 0.0001). This suggests that these two groups performed differently on the code-switched trials. That is, the English-dominant group suffered more in processing the switched sentences; while the Chinese-dominant group behaved similarly across the switch and non-switch conditions.

**Tables 3** and **4** present the results of the RTs and error rates of both groups in three different regions. Taking *region* into the model along with *language dominance* and *switch mode*, in *error analysis*, there was only a main effect of *language dominance* (*t* = 3.43, *p* < 0.001), without other main effects nor interactions. In *RT mixed-effects analysis*, three-way interactions were observed between *Regions 1 and 2* (*t* = 11, *p* < 0.0001), as well as between *Regions 2 and 3* (*t* = 10.97, *p* < 0.0001), but not between Regions 1 and 3 (*t* = 0.55, *p* = 0.58). This demonstrates that these two groups differed from each other significantly in switch effects between Regions 1 and 2, as well as between Regions 2 and 3.

**Table 3:** Mean RTs (in ms) and error rates (ERs in %) of English-dominant Bilinguals with SD in parentheses.

	Region 1 (before CS) e.g., *“my”*	Region 2 (CS) e.g., *“ 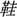 ”/“shoe”*	Region 3 (after CS) e.g., *“yesterday”*
Switch RT	680 (242)	1002 (258)	780 (236)
Non-switch RT	686 (241)	772 (222)	736 (217)
Switch ER Non-switch ER RT Difference	2.8 3 -6	3 3.2 230∗∗∗	3.5 4.3 44∗∗∗

*∗∗∗*p* < 0.001.*

**Table 4:** Mean RTs (in ms) and error rates (ERs in %) of Chinese-dominant Bilinguals with SD in parentheses.

	Region 1 (before CS) *e.g. “my”*	Region 2 (CS) *e.g. “ 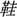 ”/“shoe”*	Region 3 (after CS) *e.g. “yesterday”*
Switch RT	802 (249)	886 (236)	951 (272)
Non-switch RT	802 (239)	945 (220)	890 (257)
Switch ER Non-switch ER Difference	6.8 8.4 0	8.6 9.2 –59∗∗∗	10.3 10.5 61∗∗∗

*∗∗∗*p* < 0.001.*

Critical comparisons should be conducted on Regions 2 and 3 separately. In critical Region 3, the mixed-effects analysis of RTs showed main effects of *language dominance* (*t* = 6.43, *p* < 0.0001) and *switch mode* (*t* = 4.43, *p* < 0.0001), but no interactions between *language dominance* and *switch mode* (*t* = 1, *p* = 0.32). These results suggest that the inhibitory effects in Region 3 were *not* modulated by *language dominance.* That is, a similar size of cost incurred when the English-dominant bilinguals switched from L2 to L1 and the Chinese-dominant bilinguals switched from L1 to L2.

In Region 2, the mixed-effects analysis of RTs showed main effects of *language dominance* (*t* = 6.51, *p* < 0.0001), and *switch mode* (*t* = 3.76, *p* < 0.001), as well as interactions between *language dominance* and *switch mode* (*t* = 14.4, *p* < 0.0001). These results suggest that *switch costs* in Region 2 were modulated by *language dominance*, with the English-dominant group producing an inhibitory effect and the Chinese-dominant group producing a facilitation effect. This pattern is consistent with the lexical activation account, due to the relative proficiency in bilinguals’ L1 and L2.

#### English-Dominant Bilinguals

The average error rate was 3.27%, and did not differ significantly across conditions. The discarded outliers comprised of 4.06% of the total trials. A delay of 230 and 44 ms was observed in Regions 2 and 3 in the switch condition respectively.

Based on current 3 (Region 1, 2, 3) × 2 (Switch vs. Non-switch) design, the overall RTs analysis in mixed-effects modeling showed that there were a main effect of *switch mode* (*t* = 11.50, *p* < 0.001), as well as main effects on *region*: Regions 1 and 2 differed significantly (*t* = 6.44, *p* < 0.001); Regions 1 and 3 differed significantly (*t* = 2.36, *p* = 0.02 < 0.05); Regions 2 and 3 differed significantly (*t* = 3.86, *p* < 0.001). These results suggest that English-dominant bilinguals encountered more difficulty in processing code-switched sentences.

Significant interactions between *switch mode* and *region* were also observed. In Region 2, the 230 ms difference across the switch and non-switch condition differed significantly from the difference in Region 1 (*t* = 13.2, *p* < 0.0001), as well as Region 3 (*t* = 9.9, *p* < 0.0001). In a similar way, the 44 ms inhibitory effect in Region 3 also differed significantly from the difference in Region 1 (*t* = 2.83, *p* = 0.005 < 0.01). Restricting the analysis to just Regions 1, 2, and 3, the mixed-effects analysis of the RTs showed significant switch costs in both Region 2 (*t* = 16.69, *p* < 0.0001), and Region 3 (*t* = 3.64, *p* < 0.001), while there was no difference in Region 1 (*t* = 0.69, *p* = 0.49). Therefore, these results show that the English-dominant bilinguals suffered from *code-switched input* in Region 2, as well as *the same input* in Region 3 when switching back. The interaction suggests that the switch cost induced in Region 2 (230 ms) was significantly larger than that in Region 3 (44 ms). In other words, switching into the weaker/less proficient language (L2) and back to the stronger/more proficient language (L1) both induced costs for the English-dominant bilinguals.

The results from the English-dominant bilinguals confirmed the presence of switch costs in reading CSs from Region 1 to Region 2 (L1–L2) and from Region 2 to Region 3 (L2–L1). The delay observed in Region 3 was unlikely to be a spill-over effect, namely, a slower response to the following word in Region 3 due to a delay in Region 2. Because the advantage of the maze task is to prevent the spill-over effect to a large extent, as discussed above (e.g., Forster et al., 2009). On the other hand, the lexical activation account is an input-driven explanation and consistent with the inhibitory effects observed in Region 2 due to the lower proficiency in L2; however, this account would not apply here in Region 3, where bilinguals were measured upon the same stimuli for both switch and non-switch conditions. Therefore, a non-lexical mechanism is required to explain the inhibitory effects in Region 3, such as the IC model.

#### Chinese-Dominant Bilinguals

The average error rate was 8.90%, and did not differ significantly across conditions. The discarded outliers comprised 12.10% of the total trials. Region 2 produced a facilitation effect of 59 ms; while Region 3 produced an inhibitory effect of 61 ms. This group of bilinguals made more errors than the English-dominant bilinguals in processing predominantly English sentences. This suggests their lower proficiency in English, corresponding to the language dominance measure.

In the same design, the overall mixed-effects analysis of the RTs showed no main effect of *switch mode* (*t* = 0.27, *p* = 0.79), unlike the English-dominant group. However, there were main effects of Region, with significant differences between Region 1 and 2 (*t* = 3.08, *p* = 0.003 < 0.01), between Region 1 and 3 (*t* = 3.71, *p* < 0.001), but no significant difference between Region 2 and 3 (*t* = 0.74, *p* = 0.46). These results suggest that the Chinese-dominant bilinguals behaved similarly across the switch and non-switch conditions in general. It is likely that the differences in Regions 2 and 3 cancel out each other.

In addition, there were significant interactions between *switch mode* and *region*: in Region 2, the 59 ms facilitation effect differed significantly from the difference (0 ms) in Region 1 (*t* = 2.91, *p* = 0.004 < 0.01), as well as the 61 ms inhibitory effect in Region 3 (*t* = 6.24, *p* < 0.0001); similarly, in Region 3, the 61 ms difference differed significantly from that in Region 1 (*t* = 3.56, *p* < 0.001) and Region 2. Restricting the analysis to just Regions 1, 2, and 3, the mixed-effects analysis of the RTs showed significant effects in both Region 2 (*t* = 4.03, *p* < 0.0001) and Region 3 (*t* = 4.46, *p* < 0.0001), while there was no difference in Region 1. These results showed that the Chinese-dominant bilinguals were faster in switching into their stronger language (L1), but slowed down when switching into the weaker one (L2).

Unlike the English-dominant group, switching from English to Chinese (L2–L1) became a facilitation effect, rather than an inhibitory effect. This result suggests that the Chinese-dominant bilinguals were faster in accessing the Chinese words than their English counterparts and the switch effect thus became facilitatory. This is consistent with the lexical activation account and in line with the pattern from the English-dominant group in Region 2, as switching to a less proficient language induced a cost while switching to a more proficient language facilitated processing.

In Region 3, inhibitory effects were observed for both groups, regardless of whether the target language (English) was the more dominant or less dominant language of the bilingual. For the same reason discussed above, the 61 ms delay was unlikely to be a spill-over effect, in addition to the fact that Region 2 elicited facilitation rather than a delay, nor could it be explained by the lexical activation account. Instead, these results confirm the presence of the switch cost observed in Region 3 in the English-dominant group and provide evidence for the inhibitory control processes involved in reading code-switches. Again, the inhibitory effects observed in Region 3 appear to be unrelated to language proficiency as switching into a more proficient or less proficient language equally slowed down the lexical processor.

## General Discussion

To summarize the results of this study, inhibitory effects were observed in Region 3 for both English-dominant and Chinese-dominant bilinguals. These results indicate that a cognitive cost incurred in reading code-switches regardless of switching to a stronger language (L2–L1) or a weaker language (L1–L2). In other words, this effect was not modulated by language dominance, unlike the switch costs reported in the production literature. On the other hand, both a facilitation effect and an inhibitory effect were observed in Region 2, which was consistent with the lexical activation account. That is, switching into a stronger/more proficient language (L2–L1) will facilitate processing; while switching into a weaker/less proficient language (L1–L2) will slow down the lexical processor. This effect was modulated by language dominance. The main novelty of the current findings lies in Region 3, as an inhibitory effect was observed in *both* directions (L1–L2 vs. L2–L1), which was not reported by previous reading studies, as far as I know.

### Language Control Effects in Region 3

In the current experimental design, the critical Region 3, where the input was the same across switch and non-switch trials, provides an ideal condition to test whether an inhibitory effect can be observed in processing code-switches. Compared to the non-switch condition (e.g., ”*my shoe yesterday*”), bilinguals encountered two switches in reading “*my 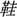 yesterday*” in the switch condition, where 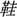 and *shoe* are translation equivalents. The cost incurred in Region 3 (e.g., *yesterday*) can only be explained by the cognitive effort to control/inhibit the other language on the previous trials (i.e., Chinese) when integrating the current trials during sentence comprehension. This effect was clearly generated by a non-lexical mechanism. These results are best explained by the inhibitory control mechanism, because an external mechanism outside the lexicon is necessary to modulate the activation level of the non-target language (i.e., the one switched from) and the target language (i.e., the one switched to). For example, the observed switch costs in Region 3 due to responses to different languages are similar in nature to task switch costs in general, even though bilinguals do not seem to encounter the selection problem in reading CSs, unlike what they do in production.

An alternative account can be proposed, however, using the BIA/BIA+ framework, which attributes switch effects to the cost of inhibiting one language node when switching to the other language. It can be assumed that bilinguals need to identify the language membership for effective reading/comprehension so that they do not access the wrong lexicon. A Chinese word would cue them to access the Chinese lexicon, while an English word would cue them to access the English one. That is, in processing CS sentences, a bilingual needs to operate two different processes: one is word identification, namely, lexical access; the other is language membership identification. A Dutch–English reader would need to wait until the completion of word identification in order to determine which language a word belongs to. This process of language membership identification is realized by activating language nodes in the BIA/BIA+ framework. Reading code-switches involves activating a different language node, as well as a different lexical representation, while inhibiting the other activated language node. This inhibition has a slower time course, producing the effects observed in Region 3 in the current study. This explanation is consistent with the BIA/BIA+ model and supports Bultena et al.’s (2014) speculation about the function of language nodes in bilingual reading. In addition, this explanation is not contradictory to the IC model, as both models implement a mechanism modulating cross-language switch costs.

However, a more distinctive orthographic feature can cue the language membership identification relatively earlier and faster in processing, as in the case with English–Chinese switches. This view is in line with the orthographic cue hypothesis proposed in Gollan et al. (1997). The idea is that the script itself provides a powerful access cue that unequivocally directs the lexical processor to a specific lexicon. Empirical evidence to support this comes from earlier masked priming work that demonstrated reliable and robust cross-script translation priming (e.g., Chinese–English in Jiang and Forster, 2001; Hebrew–English in Gollan et al., 1997, etc.) but minimal or no effects for within-script non-cognate translation priming (e.g., Dutch–English in de Groot and Nas, 1991; Spanish–English in Sanchez-Casas et al., 1992). That is, during the rapid presentation of prime-target pairs, the bilingual lexical processor could make a wrong attempt to access the wrong lexicon for the masked prime, due to the similar orthographic features of primes and targets. This can lead to null priming effects.

In other words, it is unlikely that language nodes are necessary for Chinese–English readers, as the orthography itself will cue the reader to the appropriate language membership prior to lexical access. It is inefficient for a bilingual lexical processor to activate language nodes if they are not as useful in processing. Therefore, to include a mechanism of language nodes for Chinese–English bilingual readers is not an accurate reflection of processing, unlike the Dutch–English case described in the BIA/BIA+ framework. Because they reflect CS between two different orthographic systems, the current results indicate that it is more likely that an external mechanism modulates the activation and switching of two languages, as in general task switching. As discussed above, the IC model and the BIA/BIA+ model can be complementary to each other and the current results suggest that a bilingual reading model needs to consider a mechanism that links to a general task control mechanism to explain language switching.

### Lexical Effects in Region 2

As discussed above, the behavioral data observed in Region 2 were consistent with previous reports (e.g., Bultena et al., 2014), which could be explained by the lexical activation account. That is, accessing the lexicon in a different language induced either inhibitory effects or facilitation effects, depending on the relative proficiency in the target language. According to the current results, L1–L2 switching induced a cost of 230 ms for the English-dominant bilinguals, as L2 was relatively less proficient than L1; while L2–L1 switching produced a facilitation effect of 59 ms, as activating L1 was easier than activating L2.

However, the lexical activation account, consistent with the input-driven processing mechanism illustrated by the BIA/BIA+ model, cannot rule out other mechanisms that can also impact switch costs. On the basis of the analysis in Region 3, it is important to note that the switch cost (or the reversal switch cost) incurred in Region 2 should be attributable to both the lexical effect and the language control effect. For the Chinese-dominant group, the lexical effect superseded the inhibitory effect, producing facilitation in Region 2; while the English-dominant group demonstrated slower processing in Region 2, as both the lexical effect and the language control effect were inhibitory. This analysis is consistent with previous results (e.g., Bultena et al., 2014) that demonstrated switch cost in L1–L2, but not L2–L1. It is likely that the lexical effects observed in previous studies, namely faster processing of L1, canceled out the inhibitory effects driven by the language control mechanism.

One caveat in the current design is the predictability of a return trial in Region 3 after the switched lexical items. It is possible that participants could anticipate a return trial on Region 3 right after Region 2 in a sentence after some practice. However, this expectancy strategy would *reduce* the inhibitory effects observed on Region 3 (Declerck et al., 2015), thus further supports a top–down control mechanism in processing code switches. A better design in the future research is to completely eliminate this expectancy effect.

### The Effects of Language Dominance

The effect of *language dominance* was significant across switch and non-switch conditions in Region 2. The less proficient the target language was, the higher the cost incurred in Region 2. This effect of language dominance in Region 2 is expected, consistent with the lexical activation account and previous findings. In Region 3, even though the Chinese-dominant group demonstrated slower RTs than the English-dominant group in general due to lower proficiency in English, there was no interaction between *language dominance* and *switch mode*. These results imply that switch costs in Region 3 were unrelated to language proficiency. Again, this pattern supports an inhibitory mechanism to account for the effects in Region 3.

### Inhibitory Control in Bilingual Language Comprehension

The remaining issue is the nature of the inhibitory control mechanism involved in comprehension and how it is different from that in production. Substantial empirical evidence of switch costs comes from the domain of bilingual production, as switching into a different language in production clearly involves a control mechanism that would select the intended expressions for output (e.g., Costa, 2005; Bialystok et al., 2009). In production, the control operations involve processes of maintaining a task goal, conflict monitoring, and interference suppression (Hilchey and Klein, 2011; Green and Abutalebi, 2013).

Bilingual language comprehension is driven by input and is unlikely to involve suppression of non-target language word candidates. In the process of reading CS sentences, the lexical processor encounters lexical items belonging to different language membership and responds to different language task schemas when switching. That is, switching from Language A to Language B in reading involves two processes: (1) detecting critical features that discriminate B from A (Kuipers and Thierry, 2010); and (2) controlling interference from A while activating B, similar to the notion of conflict monitoring, as the orthography of A and B are in conflict/competition in reading CSs (i.e., attending to B orthography while ignoring A orthography). The first process, as part of the word recognition process, prevents the lexical processor from accessing the wrong lexicon and thus language membership identification is necessary in bilingual reading. The cue to language membership can vary depending on how similar the input from the two languages are. In the case of reading Chinese–English switches, the orthographic cues are rather distinct and the language membership identification should be earlier and easier in the recognition process, compared with reading Dutch–English switches. The second process ensures the relative activation levels of two languages are re-settled for effective reading on a given trial. This process appears to be affected by the ability to resolve cross-language conflicts, evidenced by the inhibitory effects showed in Region 3 from both groups. Arguably, the first process is part of lexical activation, yet the locus of inhibitory effects observed in Region 3 derives from the second process, where a control mechanism is required to monitor cross-language conflicts/competition. It appears that bilingual comprehension reactively resolves language conflicts in a bottom–up manner (i.e., ignoring the irrelevant orthography while attending to the relevant orthography during language switching).

## Conclusion

This study is the first to adopt the maze task and demonstrate non-lexical inhibitory effects in the comprehension of CSs. The current results demonstrate that switch effects in reading code-switched sentences were driven by two separate mechanisms: the lexical activation (i.e., Region 2) and the inhibitory control (i.e., Region 3). In particular, the inhibitory effects in Region 3 were not modulated by language dominance, suggesting the locus of switch costs in reading derives from conflict resolution at the word form level, unlike that in production. In addition, the inhibitory effects observed in Region 3 lend support to the IC model, requiring a control mechanism external to the lexicon in language switching. However, the BIA/BIA+ framework needs to be modified to be able to explain these effects.

## Conflict of Interest Statement

The author declares that the research was conducted in the absence of any commercial or financial relationships that could be construed as a potential conflict of interest.
