# Supplementary material for: Language control in bilingual language comprehension: evidence from the maze task
Source: Front Psychol. 2015 Aug 21;6:1179. doi: 10.3389/fpsyg.2015.01179 (PMC4543796; doi:10.3389/fpsyg.2015.01179)
Supplement: Supplementary file 1 [file Supplementary_Materials.DOCX]

**Supplementary Material**

**Language Control in Bilingual Language Comprehension: evidence from the maze task**

**Xin Wang***

Department of Education, Oxford University, UK.

***Correspondence:** Xin Wang, Department of Education, Oxford University, UK.

**1. APPENDIX A**

(Questions 1 to 11 were adopted from Dunn and Fox Tree’s (2009) *Bilingual Dominance Scale*)

NAME:

MATRIC NUMBER:

1)   At what age did you first learn to speak Chinese?

| A. | 0 to 5 |
| --- | --- |
| B. | 6 to 9 |
| C. | 10 to 15 |
| D. | 16 and above |

2)   At what age did you first learn to speak English?

| A. | 0 to 5 |
| --- | --- |
| B. | 6 to 9 |
| C. | 10 to 15 |
| D. | 16 and above |

3)   At what age did you first feel comfortable speaking Chinese? If you have never felt comfortable speaking Chinese, please put no.

| A. | 0 to 5 |
| --- | --- |
| B. | 6 to 9 |
| C. | 10 to 15 |
| D. | 16 and above |
| E. | No, I have never felt comfortable speaking Chinese. |

4)   At what age did you first feel comfortable speaking English? If you have never felt comfortable speaking English, please put no.

| A. | 0 to 5 |
| --- | --- |
| B. | 6 to 9 |
| C. | 10 to 15 |
| D. | 16 and above |
| E. | No, I have never felt comfortable speaking English |

5)   Which language do you predominantly use at home?

| A. | Chinese |
| --- | --- |
| B. | English |
| C. | Both Chinese and English are frequently used |

6)

4 x 5 = ?

Which language(s) did you calculate the numbers in?

| A. | Chinese |
| --- | --- |
| B. | English |
| C. | Both Chinese and English were used in the calculation |

7)   If you had to choose either Chinese or English to use for the rest of your life, which language would it be?

| A. | Chinese |
| --- | --- |
| B. | English |

8)   How many years of schooling (primary school through university) did you have in Chinese?

| A. | 1 to 6 years |
| --- | --- |
| B. |   More than 6 years |

9)   How many years of schooling (primary school through university) did you have in English?

| A. | 1 to 6 years |
| --- | --- |
| B. | More than 6 years |

10) Do you feel that you have lost any spoken fluency in either Chinese or English? If yes, which language? At what age?

11) Do you think you have a foreign accent in either language? If so, which language?

(Foreign here implies more of unnatural, rather than non-standard.)

12)  In layman's terms, code-switching refers to the concurrent use of more than one language in conversations.

How often do you codeswitch in conversations?

| A. | I codeswitch very frequently. |
| --- | --- |
| B. |   I codeswitch quite frequently. |
| C. | I rarely codeswitch. |
| D. | I never codeswitch. |

13)   Currently, do you feel comfortable speaking English?

| A. | Yes |
| --- | --- |
| B. | No |

14)   Currently, do you feel comfortable speaking Chinese?

| A. | Yes |
| --- | --- |
| B. | No |

15)   What is your GCE ‘O’ level grade for Chinese?

| A. | A1 or A2 |
| --- | --- |
| B. | B3 or B4 |
| C. | C5 or C6 |
| D. |   Below C6 |
| E. | Have not taken before / Forgotten my grade |

16)   What is your GCE ‘O’ level grade for English?

| A. | A1 or A2 |
| --- | --- |
| B. | B3 or B4 |
| C. | C5 or C6 |
| D. | Below C6 |
| E. | Have not taken before / Forgotten my grade |

*Scoring system for Q1 – Q11 (not shown to subjects), adapted from Dunn & Fox Tree (2009) Bilingual Dominance Scale*

Q1 & Q2

Scoring: 0–5 yrs = +5, 6–9 yrs = +3, 10–15 yrs = +1, 16 and up = +0

Q3 & Q4

Scoring: 0–5 yrs = +5, 6–9 yrs = +3, 10–15 yrs = +1, 16 and up = +0,

“not yet” = +0

Q5

Scoring: if one language used at home, +5 for that language; if both used at home, +3 for each language

Q6

Scoring: +3 for language used for math; +0 if both

Q7

Scoring: +2 for language chosen for retention

Q8 & Q9

Scoring: 1–6 yrs = +1, 7 and more yrs = +2

Q10)

Scoring: −3 in language with fluency loss; −0 if neither has lost fluency

Q11)

Scoring: if one language is listed, add +5 to the opposite language of the one listed; if both languages are listed, add +3 to both languages; if no language is listed, add nothing

| **2. APPENDIX B** |  |  |  |  |  |  |
| --- | --- | --- | --- | --- | --- | --- |
|  |  |  |  |  |  |  |
|  |  |  |  |  |  |  |
| LIST A - 20 code-switched sentences & 20 non code-switched sentences | | | | | |  |
|  |  |  |  |  |  |  |
| **Correct** | **Incorrect** | **Region** |  |  |  |  |
| **Sentence Number** |  | **1** |  |  |  |  |
| This |  | 1 |  |  |  |  |
| 鸡 | 打 | 2 |  |  |  |  |
| is | we | 3 |  |  |  |  |
| so | play |  |  |  |  |  |
| oily | friend |  |  |  |  |  |
| **Sentence Number** |  | **2** |  |  |  |  |
| That |  | 1 |  |  |  |  |
| 汤 | 还 | 2 |  |  |  |  |
| is | cave | 3 |  |  |  |  |
| really | mountain |  |  |  |  |  |
| spicy | ran |  |  |  |  |  |
| **Sentence Number** |  | **3** |  |  |  |  |
| Remember |  |  |  |  |  |  |
| that | eject |  |  |  |  |  |
| the | been | 1 |  |  |  |  |
| 门 | 到 | 2 |  |  |  |  |
| is | none | 3 |  |  |  |  |
| not | could |  |  |  |  |  |
| locked | should |  |  |  |  |  |
| **Sentence Number** |  | **4** |  |  |  |  |
| The |  | 1 |  |  |  |  |
| 水 | 去 | 2 |  |  |  |  |
| is | when | 3 |  |  |  |  |
| not | which |  |  |  |  |  |
| boiled | nor |  |  |  |  |  |
| **Sentence Number** |  | **5** |  |  |  |  |
| Mum |  |  |  |  |  |  |
| said | thunder |  |  |  |  |  |
| that | ate |  |  |  |  |  |
| her | been | 1 |  |  |  |  |
| 面 | 从 | 2 |  |  |  |  |
| cost | background | 3 |  |  |  |  |
| two | worrying |  |  |  |  |  |
| bucks | through |  |  |  |  |  |
| **Sentence Number** |  | **6** |  |  |  |  |
| Their |  | 1 |  |  |  |  |
| 车 | 按 | 2 |  |  |  |  |
| is | themselves | 3 |  |  |  |  |
| over | chair |  |  |  |  |  |
| there | from |  |  |  |  |  |
| **Sentence Number** |  | **7** |  |  |  |  |
| His |  | 1 |  |  |  |  |
| 猪 | 再 | 2 |  |  |  |  |
| is | her | 3 |  |  |  |  |
| very | gloves |  |  |  |  |  |
| cute | dribbled |  |  |  |  |  |
| **Sentence Number** |  | **8** |  |  |  |  |
| This |  |  |  |  |  |  |
| leaf | heard |  |  |  |  |  |
| is | planet |  |  |  |  |  |
| green | wouldn't |  |  |  |  |  |
| and | never |  |  |  |  |  |
| that | been | 1 |  |  |  |  |
| 草 | 读 | 2 |  |  |  |  |
| is | than | 3 |  |  |  |  |
| green | drove |  |  |  |  |  |
| too | these |  |  |  |  |  |
| **Sentence Number** |  | **9** |  |  |  |  |
| That |  | 1 |  |  |  |  |
| 肉 | 唱 | 2 |  |  |  |  |
| was | shout | 3 |  |  |  |  |
| indeed | were |  |  |  |  |  |
| overcooked | which |  |  |  |  |  |
| **Sentence Number** |  | **10** |  |  |  |  |
| His |  | 1 |  |  |  |  |
| 狗 | 我 | 2 |  |  |  |  |
| bit | forest | 3 |  |  |  |  |
| my | swims |  |  |  |  |  |
| finger | observe |  |  |  |  |  |
| **Sentence Number** |  | **11** |  |  |  |  |
| All |  |  |  |  |  |  |
| the | him | 1 |  |  |  |  |
| money | shut | 2 |  |  |  |  |
| is | blanket | 3 |  |  |  |  |
| gone | and |  |  |  |  |  |
| **Sentence Number** |  | **12** |  |  |  |  |
| The |  | 1 |  |  |  |  |
| fire | out | 2 |  |  |  |  |
| is | an | 3 |  |  |  |  |
| getting | which |  |  |  |  |  |
| bigger | sneeze |  |  |  |  |  |
| **Sentence Number** |  | **13** |  |  |  |  |
| Its |  | 1 |  |  |  |  |
| fur | and | 2 |  |  |  |  |
| is | sea | 3 |  |  |  |  |
| really | apply |  |  |  |  |  |
| dropping | however |  |  |  |  |  |
| **Sentence Number** |  | **14** |  |  |  |  |
| My |  |  |  |  |  |  |
| tummy | dreamt |  |  |  |  |  |
| hurts | seven |  |  |  |  |  |
| and | pork |  |  |  |  |  |
| my | or | 1 |  |  |  |  |
| hand | it | 2 |  |  |  |  |
| is | army | 3 |  |  |  |  |
| bleeding | fever |  |  |  |  |  |
| **Sentence Number** |  | **15** |  |  |  |  |
| Her |  |  |  |  |  |  |
| wallet | drank |  |  |  |  |  |
| is | nor |  |  |  |  |  |
| missing | screamed |  |  |  |  |  |
| and | was |  |  |  |  |  |
| her | or | 1 |  |  |  |  |
| leg | moved | 2 |  |  |  |  |
| is | himself | 3 |  |  |  |  |
| injured | thanks |  |  |  |  |  |
| **Sentence Number** |  | **16** |  |  |  |  |
| Our |  | 1 |  |  |  |  |
| book | take | 2 |  |  |  |  |
| is | cow | 3 |  |  |  |  |
| with | or |  |  |  |  |  |
| him | sat |  |  |  |  |  |
| **Sentence Number** |  | **17** |  |  |  |  |
| Their |  | 1 |  |  |  |  |
| gun | not | 2 |  |  |  |  |
| looks | chairs | 3 |  |  |  |  |
| like | took |  |  |  |  |  |
| a | wrote |  |  |  |  |  |
| toy | although |  |  |  |  |  |
| **Sentence Number** |  | **18** |  |  |  |  |
| The |  | 1 |  |  |  |  |
| trees | brings | 2 |  |  |  |  |
| provide | black | 3 |  |  |  |  |
| good | accelerate |  |  |  |  |  |
| shade | and |  |  |  |  |  |
| **Sentence Number** |  | **19** |  |  |  |  |
| The |  | 1 |  |  |  |  |
| cup | down | 2 |  |  |  |  |
| needs | blue | 3 |  |  |  |  |
| to | drank |  |  |  |  |  |
| be | so |  |  |  |  |  |
| washed | when |  |  |  |  |  |
| **Sentence Number** |  | **20** |  |  |  |  |
| That |  | 1 |  |  |  |  |
| cat | she | 2 |  |  |  |  |
| looks | kilogram | 3 |  |  |  |  |
| pretty | nudge |  |  |  |  |  |
| harmless | fell |  |  |  |  |  |
| **Sentence Number** |  | **21** |  |  |  |  |
| David |  |  |  |  |  |  |
| threw | heavy |  |  |  |  |  |
| a | or | 1 |  |  |  |  |
| 球 | 九 | 2 |  |  |  |  |
| towards | friendly | 3 |  |  |  |  |
| me | maybe |  |  |  |  |  |
| **Sentence Number** |  | **22** |  |  |  |  |
| Dad |  |  |  |  |  |  |
| fried | white |  |  |  |  |  |
| the | listen | 1 |  |  |  |  |
| 蛋 | 过 | 2 |  |  |  |  |
| for | brick | 3 |  |  |  |  |
| her | stopped |  |  |  |  |  |
| **Sentence Number** |  | **23** |  |  |  |  |
| I |  |  |  |  |  |  |
| saw | out |  |  |  |  |  |
| his | if | 1 |  |  |  |  |
| 牛 | 吃 | 2 |  |  |  |  |
| over | an | 3 |  |  |  |  |
| there | thorough |  |  |  |  |  |
| **Sentence Number** |  | **24** |  |  |  |  |
| The |  |  |  |  |  |  |
| maid | then |  |  |  |  |  |
| brewed | been |  |  |  |  |  |
| some | cornered | 1 |  |  |  |  |
| 茶 | 你 | 2 |  |  |  |  |
| for | look | 3 |  |  |  |  |
| me | faint |  |  |  |  |  |
| **Sentence Number** |  | **25** |  |  |  |  |
| This |  |  |  |  |  |  |
| thing | within |  |  |  |  |  |
| can | angry |  |  |  |  |  |
| save | of |  |  |  |  |  |
| a | sleeping |  |  |  |  |  |
| lot | cannot |  |  |  |  |  |
| of | palm | 1 |  |  |  |  |
| 电 | 有 | 2 |  |  |  |  |
| for | take | 3 |  |  |  |  |
| the | lot |  |  |  |  |  |
| company | into |  |  |  |  |  |
| **Sentence Number** |  | **26** |  |  |  |  |
| The |  |  |  |  |  |  |
| lock | therefore |  |  |  |  |  |
| is | this |  |  |  |  |  |
| made | are |  |  |  |  |  |
| of | worked | 1 |  |  |  |  |
| 铁 | 游 | 2 |  |  |  |  |
| **Sentence Number** |  | **27** |  |  |  |  |
| I |  |  |  |  |  |  |
| bought | of |  |  |  |  |  |
| some | pushed | 1 |  |  |  |  |
| 菜 | 不 | 2 |  |  |  |  |
| from | see | 3 |  |  |  |  |
| the | ticked |  |  |  |  |  |
| market | and |  |  |  |  |  |
| **Sentence Number** |  | **28** |  |  |  |  |
| He |  |  |  |  |  |  |
| stared | cubic |  |  |  |  |  |
| at | red |  |  |  |  |  |
| the | to | 1 |  |  |  |  |
| 云 | 买 | 2 |  |  |  |  |
| **Sentence Number** |  | **29** |  |  |  |  |
| The |  |  |  |  |  |  |
| robbers | to |  |  |  |  |  |
| punched | cruel |  |  |  |  |  |
| her | drowned | 1 |  |  |  |  |
| 头 | 卖 | 2 |  |  |  |  |
| and | surely | 3 |  |  |  |  |
| kicked | or |  |  |  |  |  |
| her | might |  |  |  |  |  |
| **Sentence Number** |  | **30** |  |  |  |  |
| I |  |  |  |  |  |  |
| bought | shy |  |  |  |  |  |
| the | of | 1 |  |  |  |  |
| 花 | 西 | 2 |  |  |  |  |
| for | rivers | 3 |  |  |  |  |
| her | rarely |  |  |  |  |  |
| **Sentence Number** |  | **31** |  |  |  |  |
| He |  |  |  |  |  |  |
| climbed | her |  |  |  |  |  |
| over | pumped |  |  |  |  |  |
| that | under | 1 |  |  |  |  |
| wall | run | 2 |  |  |  |  |
| easily | he | 3 |  |  |  |  |
| **Sentence Number** |  | **32** |  |  |  |  |
| Sharpen |  |  |  |  |  |  |
| this | played | 1 |  |  |  |  |
| knife | in | 2 |  |  |  |  |
| before | view | 3 |  |  |  |  |
| using | then |  |  |  |  |  |
| **Sentence Number** |  | **33** |  |  |  |  |
| My |  |  |  |  |  |  |
| martial | than |  |  |  |  |  |
| arts | when |  |  |  |  |  |
| instructor | my |  |  |  |  |  |
| likes | although |  |  |  |  |  |
| that | sought | 1 |  |  |  |  |
| sword | this | 2 |  |  |  |  |
| **Sentence Number** |  | **34** |  |  |  |  |
| She |  |  |  |  |  |  |
| ate | creative |  |  |  |  |  |
| the | than | 1 |  |  |  |  |
| rice | that | 2 |  |  |  |  |
| so | drank | 3 |  |  |  |  |
| quickly | mouse |  |  |  |  |  |
| **Sentence Number** |  | **35** |  |  |  |  |
| Put |  |  |  |  |  |  |
| some | drew | 1 |  |  |  |  |
| ice | ten | 2 |  |  |  |  |
| in | lure | 3 |  |  |  |  |
| the | tore |  |  |  |  |  |
| box | seen |  |  |  |  |  |
| **Sentence Number** |  | **36** |  |  |  |  |
| Don't |  |  |  |  |  |  |
| lie | shall |  |  |  |  |  |
| on | maybe |  |  |  |  |  |
| her | under | 1 |  |  |  |  |
| bed | does | 2 |  |  |  |  |
| **Sentence Number** |  | **37** |  |  |  |  |
| Close |  |  |  |  |  |  |
| the | give | 1 |  |  |  |  |
| window | write | 2 |  |  |  |  |
| before | however | 3 |  |  |  |  |
| it | painted |  |  |  |  |  |
| rains | fake |  |  |  |  |  |
| **Sentence Number** |  | **38** |  |  |  |  |
| Don't |  |  |  |  |  |  |
| add | then |  |  |  |  |  |
| so | is |  |  |  |  |  |
| much | verb | 1 |  |  |  |  |
| oil | walk | 2 |  |  |  |  |
| **Sentence Number** |  | **39** |  |  |  |  |
| Apply |  |  |  |  |  |  |
| some | don't | 1 |  |  |  |  |
| powder | throw | 2 |  |  |  |  |
| before | know | 3 |  |  |  |  |
| you | or |  |  |  |  |  |
| sleep | the |  |  |  |  |  |
| **Sentence Number** |  | **40** |  |  |  |  |
| I |  |  |  |  |  |  |
| polished | thus |  |  |  |  |  |
| my | drew | 1 |  |  |  |  |
| shoes | think | 2 |  |  |  |  |
| yesterday | sad | 3 |  |  |  |  |
|  |  |  |  |  |  |  |
|  |  |  |  |  |  |  |
| LIST B - 20 code-switched sentences & 20 non code-switched sentences | | | | | |  |
|  |  |  |  |  |  |  |
| **Correct** | **Incorrect** | **Region** |  |  |  |  |
| **Sentence Number** |  | **1** |  |  |  |  |
| This |  | 1 |  |  |  |  |
| chicken | hit | 2 |  |  |  |  |
| is | we | 3 |  |  |  |  |
| so | play |  |  |  |  |  |
| oily | friend |  |  |  |  |  |
| **Sentence Number** |  | **2** |  |  |  |  |
| That |  | 1 |  |  |  |  |
| soup | yet | 2 |  |  |  |  |
| is | cave | 3 |  |  |  |  |
| really | mountain |  |  |  |  |  |
| spicy | ran |  |  |  |  |  |
| **Sentence Number** |  | **3** |  |  |  |  |
| Remember |  |  |  |  |  |  |
| that | eject |  |  |  |  |  |
| the | been | 1 |  |  |  |  |
| door | to | 2 |  |  |  |  |
| is | none | 3 |  |  |  |  |
| not | could |  |  |  |  |  |
| locked | should |  |  |  |  |  |
| **Sentence Number** |  | **4** |  |  |  |  |
| The |  | 1 |  |  |  |  |
| water | go | 2 |  |  |  |  |
| is | when | 3 |  |  |  |  |
| not | which |  |  |  |  |  |
| boiled | nor |  |  |  |  |  |
| **Sentence Number** |  | **5** |  |  |  |  |
| Mum |  |  |  |  |  |  |
| said | thunder |  |  |  |  |  |
| that | ate |  |  |  |  |  |
| her | been | 1 |  |  |  |  |
| noodles | from | 2 |  |  |  |  |
| cost | background | 3 |  |  |  |  |
| two | worrying |  |  |  |  |  |
| bucks | through |  |  |  |  |  |
| **Sentence Number** |  | **6** |  |  |  |  |
| Their |  | 1 |  |  |  |  |
| car | press | 2 |  |  |  |  |
| is | themselves | 3 |  |  |  |  |
| over | chair |  |  |  |  |  |
| there | from |  |  |  |  |  |
| **Sentence Number** |  | **7** |  |  |  |  |
| His |  | 1 |  |  |  |  |
| pig | again | 2 |  |  |  |  |
| is | her | 3 |  |  |  |  |
| very | gloves |  |  |  |  |  |
| cute | dribbled |  |  |  |  |  |
| **Sentence Number** |  | **8** |  |  |  |  |
| This |  |  |  |  |  |  |
| leaf | heard |  |  |  |  |  |
| is | planet |  |  |  |  |  |
| green | wouldn't |  |  |  |  |  |
| and | never |  |  |  |  |  |
| that | been | 1 |  |  |  |  |
| grass | read | 2 |  |  |  |  |
| is | than | 3 |  |  |  |  |
| green | drove |  |  |  |  |  |
| too | these |  |  |  |  |  |
| **Sentence Number** |  | **9** |  |  |  |  |
| That |  | 1 |  |  |  |  |
| meat | sing | 2 |  |  |  |  |
| was | shout | 3 |  |  |  |  |
| indeed | were |  |  |  |  |  |
| overcooked | which |  |  |  |  |  |
| **Sentence Number** |  | **10** |  |  |  |  |
| His |  | 1 |  |  |  |  |
| dog | me | 2 |  |  |  |  |
| bit | forest | 3 |  |  |  |  |
| my | swims |  |  |  |  |  |
| finger | observe |  |  |  |  |  |
| **Sentence Number** |  | **11** |  |  |  |  |
| All |  |  |  |  |  |  |
| the | him | 1 |  |  |  |  |
| 钱 | 关 | 2 |  |  |  |  |
| is | blanket | 3 |  |  |  |  |
| gone | and |  |  |  |  |  |
| **Sentence Number** |  | **12** |  |  |  |  |
| The |  | 1 |  |  |  |  |
| 火 | 出 | 2 |  |  |  |  |
| is | an | 3 |  |  |  |  |
| getting | which |  |  |  |  |  |
| bigger | sneeze |  |  |  |  |  |
| **Sentence Number** |  | **13** |  |  |  |  |
| Its |  | 1 |  |  |  |  |
| 毛 | 和 | 2 |  |  |  |  |
| is | sea | 3 |  |  |  |  |
| really | apply |  |  |  |  |  |
| dropping | cry |  |  |  |  |  |
| **Sentence Number** |  | **14** |  |  |  |  |
| My |  |  |  |  |  |  |
| tummy | dreamt |  |  |  |  |  |
| hurts | seven |  |  |  |  |  |
| and | pork |  |  |  |  |  |
| my | or | 1 |  |  |  |  |
| 手 | 它 | 2 |  |  |  |  |
| is | army | 3 |  |  |  |  |
| bleeding | fever |  |  |  |  |  |
| **Sentence Number** |  | **15** |  |  |  |  |
| Her |  |  |  |  |  |  |
| wallet | drank |  |  |  |  |  |
| is | nor |  |  |  |  |  |
| missing | screamed |  |  |  |  |  |
| and | was |  |  |  |  |  |
| her | or | 1 |  |  |  |  |
| 脚 | 动 | 2 |  |  |  |  |
| is | himself | 3 |  |  |  |  |
| injured | thanks |  |  |  |  |  |
| **Sentence Number** |  | **16** |  |  |  |  |
| Our |  | 1 |  |  |  |  |
| 书 | 取 | 2 |  |  |  |  |
| is | cow | 3 |  |  |  |  |
| with | or |  |  |  |  |  |
| him | sat |  |  |  |  |  |
| **Sentence Number** |  | **17** |  |  |  |  |
| Their |  | 1 |  |  |  |  |
| 枪 | 没 | 2 |  |  |  |  |
| looks | chairs | 3 |  |  |  |  |
| like | took |  |  |  |  |  |
| a | wrote |  |  |  |  |  |
| toy | although |  |  |  |  |  |
| **Sentence Number** |  | **18** |  |  |  |  |
| The |  | 1 |  |  |  |  |
| 树 | 拿 | 2 |  |  |  |  |
| provide | black | 3 |  |  |  |  |
| good | accelerate |  |  |  |  |  |
| shade | and |  |  |  |  |  |
| **Sentence Number** |  | **19** |  |  |  |  |
| The |  | 1 |  |  |  |  |
| 杯 | 下 | 2 |  |  |  |  |
| needs | blue | 3 |  |  |  |  |
| to | drank |  |  |  |  |  |
| be | so |  |  |  |  |  |
| washed | when |  |  |  |  |  |
| **Sentence Number** |  | **20** |  |  |  |  |
| That |  | 1 |  |  |  |  |
| 猫 | 她 | 2 |  |  |  |  |
| looks | kilogram | 3 |  |  |  |  |
| pretty | nudge |  |  |  |  |  |
| harmless | fell |  |  |  |  |  |
| **Sentence Number** |  | **21** |  |  |  |  |
| David |  |  |  |  |  |  |
| threw | heavy |  |  |  |  |  |
| a | or | 1 |  |  |  |  |
| ball | nine | 2 |  |  |  |  |
| towards | friendly | 3 |  |  |  |  |
| me | maybe |  |  |  |  |  |
| **Sentence Number** |  | **22** |  |  |  |  |
| Dad |  |  |  |  |  |  |
| fried | white |  |  |  |  |  |
| the | listen | 1 |  |  |  |  |
| egg | across | 2 |  |  |  |  |
| for | brick | 3 |  |  |  |  |
| her | stopped |  |  |  |  |  |
| **Sentence Number** |  | **23** |  |  |  |  |
| I |  |  |  |  |  |  |
| saw | out |  |  |  |  |  |
| his | if | 1 |  |  |  |  |
| cow | eat | 2 |  |  |  |  |
| over | an | 3 |  |  |  |  |
| there | thorough |  |  |  |  |  |
| **Sentence Number** |  | **24** |  |  |  |  |
| The |  |  |  |  |  |  |
| maid | then |  |  |  |  |  |
| brewed | been |  |  |  |  |  |
| some | cornered | 1 |  |  |  |  |
| tea | you | 2 |  |  |  |  |
| for | look | 3 |  |  |  |  |
| me | faint |  |  |  |  |  |
| **Sentence Number** |  | **25** |  |  |  |  |
| This |  |  |  |  |  |  |
| thing | within |  |  |  |  |  |
| can | angry |  |  |  |  |  |
| save | of |  |  |  |  |  |
| a | sleeping |  |  |  |  |  |
| lot | cannot |  |  |  |  |  |
| of | palm | 1 |  |  |  |  |
| electricity | have | 2 |  |  |  |  |
| for | take | 3 |  |  |  |  |
| the | lot |  |  |  |  |  |
| company | into |  |  |  |  |  |
| **Sentence Number** |  | **26** |  |  |  |  |
| The |  |  |  |  |  |  |
| lock | therefore |  |  |  |  |  |
| is | this |  |  |  |  |  |
| made | are |  |  |  |  |  |
| of | worked | 1 |  |  |  |  |
| iron | swim | 2 |  |  |  |  |
| **Sentence Number** |  | **27** |  |  |  |  |
| I |  |  |  |  |  |  |
| bought | of |  |  |  |  |  |
| some | pushed | 1 |  |  |  |  |
| vegetables | no | 2 |  |  |  |  |
| from | see | 3 |  |  |  |  |
| the | ticked |  |  |  |  |  |
| market | and |  |  |  |  |  |
| **Sentence Number** |  | **28** |  |  |  |  |
| He |  |  |  |  |  |  |
| stared | cubic |  |  |  |  |  |
| at | red |  |  |  |  |  |
| the | to | 1 |  |  |  |  |
| clouds | buy | 2 |  |  |  |  |
| **Sentence Number** |  | **29** |  |  |  |  |
| The |  |  |  |  |  |  |
| robbers | to |  |  |  |  |  |
| punched | cruel |  |  |  |  |  |
| her | drowned | 1 |  |  |  |  |
| head | sell | 2 |  |  |  |  |
| and | surely | 3 |  |  |  |  |
| kicked | or |  |  |  |  |  |
| her | might |  |  |  |  |  |
| **Sentence Number** |  | **30** |  |  |  |  |
| I |  |  |  |  |  |  |
| bought | shy |  |  |  |  |  |
| the | of | 1 |  |  |  |  |
| flowers | west | 2 |  |  |  |  |
| for | rivers | 3 |  |  |  |  |
| her | rarely |  |  |  |  |  |
| **Sentence Number** |  | **31** |  |  |  |  |
| He |  |  |  |  |  |  |
| climbed | her |  |  |  |  |  |
| over | pumped |  |  |  |  |  |
| that | under | 1 |  |  |  |  |
| 墙 | 跑 | 2 |  |  |  |  |
| easily | he | 3 |  |  |  |  |
| **Sentence Number** |  | **32** |  |  |  |  |
| Sharpen |  |  |  |  |  |  |
| this | played | 1 |  |  |  |  |
| 刀 | 里 | 2 |  |  |  |  |
| before | view | 3 |  |  |  |  |
| using | then |  |  |  |  |  |
| **Sentence Number** |  | **33** |  |  |  |  |
| My |  |  |  |  |  |  |
| martial | than |  |  |  |  |  |
| arts | when |  |  |  |  |  |
| instructor | my |  |  |  |  |  |
| likes | above |  |  |  |  |  |
| that | sought | 1 |  |  |  |  |
| 剑 | 这 | 2 |  |  |  |  |
| **Sentence Number** |  | **34** |  |  |  |  |
| She |  |  |  |  |  |  |
| ate | creative |  |  |  |  |  |
| the | than | 1 |  |  |  |  |
| 饭 | 那 | 2 |  |  |  |  |
| so | drank | 3 |  |  |  |  |
| quickly | mouse |  |  |  |  |  |
| **Sentence Number** |  | **35** |  |  |  |  |
| Put |  |  |  |  |  |  |
| some | drew | 1 |  |  |  |  |
| 冰 | 十 | 2 |  |  |  |  |
| in | lure | 3 |  |  |  |  |
| the | tore |  |  |  |  |  |
| box | seen |  |  |  |  |  |
| **Sentence Number** |  | **36** |  |  |  |  |
| Don't |  |  |  |  |  |  |
| lie | shall |  |  |  |  |  |
| on | maybe |  |  |  |  |  |
| her | under | 1 |  |  |  |  |
| 床 | 做 | 2 |  |  |  |  |
| **Sentence Number** |  | **37** |  |  |  |  |
| Close |  |  |  |  |  |  |
| the | give | 1 |  |  |  |  |
| 窗 | 写 | 2 |  |  |  |  |
| before | however | 3 |  |  |  |  |
| it | painted |  |  |  |  |  |
| rains | fake |  |  |  |  |  |
| **Sentence Number** |  | **38** |  |  |  |  |
| Don't |  |  |  |  |  |  |
| add | then |  |  |  |  |  |
| so | is |  |  |  |  |  |
| much | verb | 1 |  |  |  |  |
| 油 | 走 | 2 |  |  |  |  |
| **Sentence Number** |  | **39** |  |  |  |  |
| Apply |  |  |  |  |  |  |
| some | don't | 1 |  |  |  |  |
| 粉 | 丢 | 2 |  |  |  |  |
| before | know | 3 |  |  |  |  |
| you | or |  |  |  |  |  |
| sleep | the |  |  |  |  |  |
| **Sentence Number** |  | **40** |  |  |  |  |
| I |  |  |  |  |  |  |
| polished | thus |  |  |  |  |  |
| my | drew | 1 |  |  |  |  |
| 鞋 | 想 | 2 |  |  |  |  |
| yesterday | sad | 3 |  |  |  |  |
|  |  |  |  |  |  |  |
|  |  |  |  |  |  |  |
|  |  |  |  |  |  |  |

**3. APPENDIX C**

These are some of the Chinese characters that appeared in the word-maze task. If there are any words that you do not know, please indicate an ‘**X**’ beside it.

If you know all of the words in this list, leave the list blank.

| 鸡 | 打 | 球 | 九 | 红 | 八 |
| --- | --- | --- | --- | --- | --- |
| 汤 | 还 | 蛋 | 过 | 忍 | 金 |
| 门 | 到 | 牛 | 可 | 哭 | 么 |
| 水 | 去 | 茶 | 你 | 笨 | 们 |
| 面 | 从 | 电 | 有 | 美 | 您 |
| 车 | 按 | 铁 | 游 | 笑 | 大 |
| 猪 | 再 | 菜 | 不 | 胖 | 为 |
| 草 | 读 | 云 | 买 | 坐 | 雨 |
| 肉 | 唱 | 头 | 卖 | 跳 | 也 |
| 狗 | 我 | 花 | 西 | 忙 | 当 |
| 钱 | 关 | 墙 | 跑 | 热 | 本 |
| 火 | 出 | 刀 | 里 | 叫 | 黄 |
| 毛 | 和 | 剑 | 这 | 杯 | 下 |
| 手 | 它 | 饭 | 那 | 闷 | 人 |
| 脚 | 动 | 冰 | 十 | 小鸟 | 可是 |
| 书 | 取 | 床 | 做 | 粉 | 丢 |
| 枪 | 没 | 窗 | 写 | 鞋 | 想 |
| 树 | 拿 | 油 | 走 | 猫 | 她 |
| 玩具 | 上学 |  |  |  |  |
